# The Impact of Nutrients on Mental Health and Well-Being: Insights From the Literature

**DOI:** 10.3389/fnut.2021.656290

**Published:** 2021-03-08

**Authors:** Maurizio Muscaritoli

**Affiliations:** Department of Translational and Precision Medicine, Sapienza University of Rome, Rome, Italy

**Keywords:** eicosapentaenoic acid, docosahexaenoic acid, alpha-tocopherol, magnesium, folic acid, mental health, well-being, inflammation

## Abstract

A good nutritional status is important for maintaining normal body function and preventing or mitigating the dysfunction induced by internal or external factors. Nutritional deficiencies often result in impaired function, and, conversely, intakes at recommended levels can resume or further enhance body functions. An increasing number of studies are revealing that diet and nutrition are critical not only for physiology and body composition, but also have significant effects on mood and mental well-being. In particular, Western dietary habits have been the object of several research studies focusing on the relationship between nutrition and mental health. This review aims to summarize the current knowledge about the relationship between the intake of specific micro- and macronutrients, including eicosapentaenoic acid, docosahexaenoic acid, alpha-tocopherol, magnesium and folic acid, and mental health, with particular reference to their beneficial effect on stress, sleep disorders, anxiety, mild cognitive impairment, as well as on neuropsychiatric disorders, all significantly affecting the quality of life of an increasing number of people. Overall data support a positive role for the nutrients mentioned above in the preservation of normal brain function and mental well-being, also through the control of neuroinflammation, and encourage their integration in a well-balanced and varied diet, accompanied by a healthy lifestyle. This strategy is of particular importance when considering the global human aging and that the brain suffers significantly from the life-long impact of stress factors.

## Introduction

**Figure 1:**
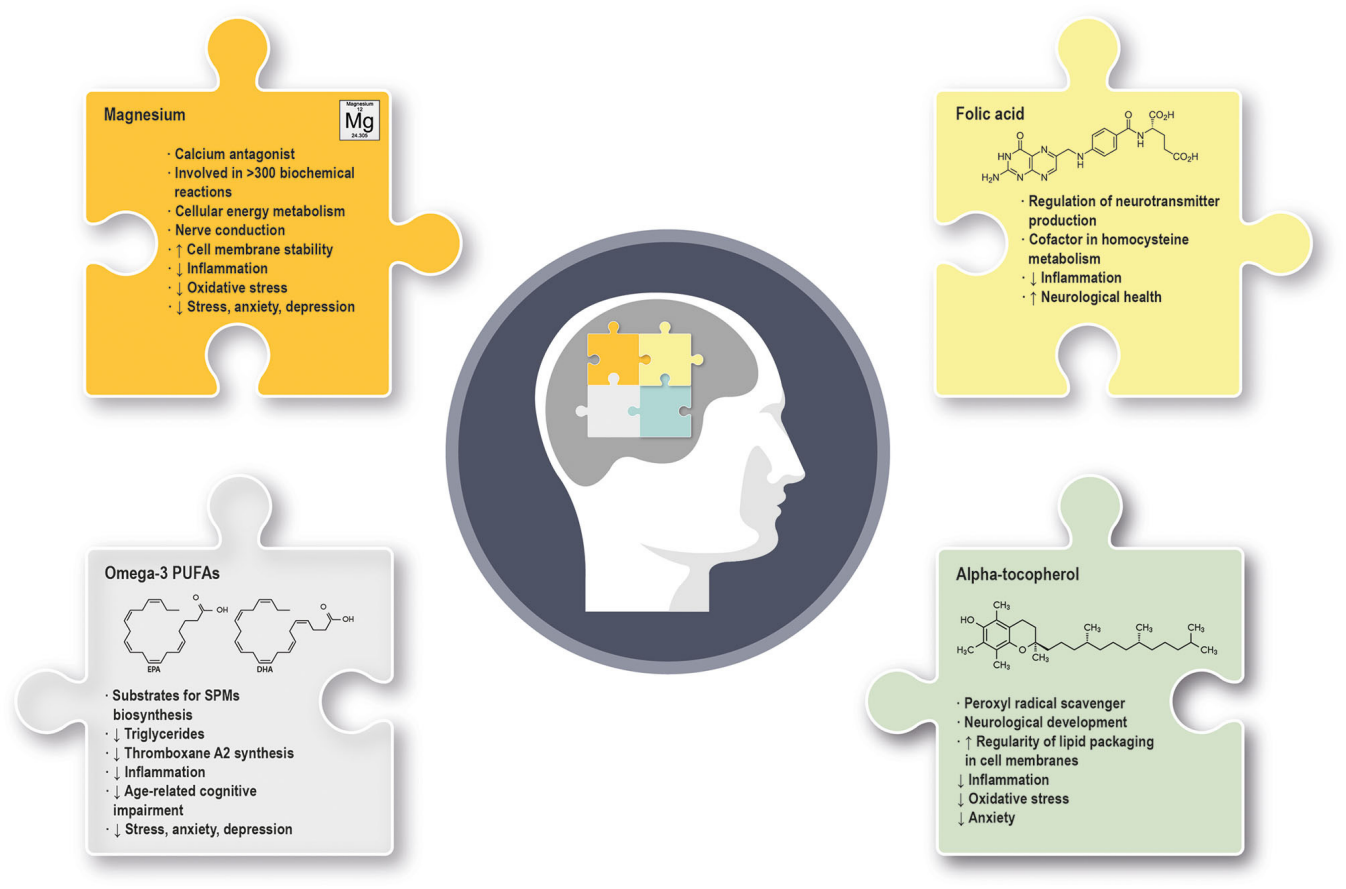
Summary of the main molecular and physiological effects exerted by omega-3 PUFAs, alpha-tocopherol, magnesium and folic acid in mental health and well-being. (↓) indicates downregulation; (↑) indicates upregulation. PUFAs, polyunsaturated fatty acids; SPMs, specialized pro-resolving mediators.

A healthy dietary pattern can affect mental health and well-being through anti-inflammatory, antioxidant, neurogenesis, microbiome- and immune-modifying mechanisms, as well as through epigenetic modifications (1). Dietary profile affects not only the brain composition, structure and function, but also endogenous hormones, neuropeptides, neurotransmitters, and the microbiota-gut-brain axis, in turn playing a key role in modulation of stress and inflammation and in preservation of cognitive function (2). In addition to a healthy and balanced diet, the supplementation of micronutrients (e.g., vitamins and minerals) and macronutrients (e.g., fatty acids) can provide several beneficial effects, due to their multiple biological roles (3). This narrative review aims to summarize the current knowledge about the relationship between the intake of specific nutrients, including the omega-3 polyunsaturated fatty acids (PUFAs) eicosapentaenoic acid (EPA) and docosahexaenoic acid (DHA), alpha-tocopherol, magnesium and folic acid, and the beneficial effect on mental health and well-being (Figure 1). A comprehensive search and critical review have been conducted in PubMed database using the keywords stress, anxiety, sleep disorders, mild cognitive impairment (MCI), depression, bipolar disorders, obsessive-compulsive disorder, neuroinflammation, inflammation, associated with the nutrients alpha-tocopherol, folic acid/folate, magnesium, omega-3 fatty acids, EPA, and DHA through the use of the Boolean operators AND, OR, identifying the articles relevant to this review. The literature search has been limited to English language articles. Additionally, manual searches for related articles have also been performed. The time range for literature search has been 1994–2020, with about 70% of articles published in 2016–2020. Randomized clinical trials (RCTs) have been prioritized in the selection of research studies to be reviewed; however, some pre-clinical studies in animal models of disease have been discussed as well.

EPA and DHA are the predominant PUFAs, are endowed with anti-inflammatory properties and exert various biological activities, mainly as substrates for the biosynthesis of specialized pro-resolving mediators (SPMs) and as agonists of cellular receptors (4). DHA is highly concentrated in the central nervous system (CNS), where it plays a key role in optimal development and later cognitive functioning (5). Conversely, steady-state concentrations of EPA are low in the brain, due to its rapid metabolism (6). DHA decreases in the brain during aging and this should be of particular concern in Western countries, where the diet might be poor of omega-3 PUFAs (7). Of interest, EPA and DHA can be converted in their respective endocannabinoid derivatives, eicosapentaenoyl-ethanolamine (EPEA), and docosahexaenoyl-ethanolamine (DHEA), with, among others, potential antidepressive effects (8).

Alpha-tocopherol is one of the eight isoforms of vitamin E, located mainly in cell membranes, where it increases the regularity of lipid packaging. Alpha-tocopherol exerts a protective antioxidant and anti-inflammatory activity that efficiently prevents lipid peroxidation. It accumulates particularly at sites where free radical production is higher, such as the mitochondrial membranes. Alpha-tocopherol is essential for neurological development (9) and is one of the most effective nutrients known to modulate the immune function. A deficiency of vitamin E can occur in the setting of chronic fat malabsorption or lipoprotein deficiency, or derive from low dietary amounts, more frequently in children than in adults. Alpha-tocopherol deficiency impairs both the innate and the adaptive immune function (10), can increase adverse events during pregnancy and lead to neurological abnormalities or disorders (11).

Folic acid is the synthetic form of the water-soluble vitamin B9, also known as folate. Both folate and folic acid must be metabolized into the more bioavailable form L-methylfolate to play their several roles. L-methylfolate is able to cross the blood–brain barrier (BBB) and regulate the production of the neurotransmitters dopamine, norepinephrine and serotonin, thus contributing to mental function and performance (12). Several B-vitamins, including the B9, are also cofactors in homocysteine metabolism and are able to reduce inflammation caused by high levels of this amino acid (13). Folic acid deficiency is caused mainly by inadequate dietary intake and can lead to neurological symptoms in adults and to neural tube defects in the fetus (3).

Magnesium is the second most abundant intracellular cation in human body, acts as calcium antagonist and has a wide range of biological roles, including cellular energy metabolism, nerve conduction, membrane stability, and synaptic transmission. Magnesium has also anti-inflammatory properties mediated by the reduced expression and release of pro-inflammatory molecules (14). Hypomagnesemia usually occurs in critically ill patients due to gastrointestinal loss or dysregulation of renal reabsorption. Also, certain drugs and alcohol use can cause hypomagnesemia (3). Of note, hypomagnesemia can be frequently due to the soil used for agriculture and to extensive intake of processed food and refined grains (15).

## Nutrition and Mental Health Preservation or Restoration: Can Omega-3 Fatty Acids, Alpha- Tocopherol, Magnesium and Folic Acid Affect Stress, Anxiety, Sleep Disorders and Mild Cognitive Impairment?

### Potential Effects on Stress

Stress is a body reaction to the disturbance of a status of equilibrium. Stress contributes to an overall reduction of the quality of life (QoL) and to a wide range of disorders, including hypertension, cardiovascular disease, inflammatory bowel syndrome, diabetes mellitus (16). In particular, stress can lead to a prolonged release of cortisol and subsequent activation of immune cells, especially neutrophils, followed by the production of free radicals (17, 18). Nutritional imbalance, insufficient vitamin intake, and excessive consumption of fat can result in dysregulation of stress hormones and inflammation (16). Conversely, a healthy nutrition can greatly contribute to bridge over the stress and reduce the probability of stress-related disease. In this setting, a RCT supported a positive impact of folate and magnesium oxide supplementation on pro-inflammatory cytokine profiles in subjects experiencing stress, but without significant changes of psychological measures (19). Magnesium supplementation alleviated stress in healthy adults with hypomagnesemia in a phase IV RCT (20). The administration of DHA to stressed healthy subjects in a RCT resulted in a significant reduction of perceived stress, supporting the protective or “adaptogenic” role of omega-3 PUFAs (21). Omega-3 PUFAs also prevented cortisol increase and reduced the symptoms of occupational burnout in a double-blind RCT (22) (Table 1). Significant evidences for an association between alpha-tocopherol levels and stress are still lacking in humans.

**Table 1:** Summary of the clinical trials we reviewed and providing statistically significant results about the beneficial effect of nutrient supplementation on mental health and well-being.

**Tested molecule(s)**	**Disorder**	**Study Design**	**Number of participants**	**Main results**	**Reference**
Magnesium oxide, folic acid, multivitamin supplementation	Stress	8-week randomized controlled trial on women aged 25–45 and experiencing psychological distress. Post-intervention data collected 8 weeks after the start of the supplement intake	60 subjects randomized in an active group or in a placebo group	Positive impact of nutrient supplementation on pro-inflammatory cytokine profiles in the active group; no effect on psychological state	(19)
Magnesium, vitamin B6	Stress	8-week, Phase IV, randomized, controlled, investigator-blinded, parallel-group trial on subjects aged 18 to 50, with moderate to extremely severe stress and hypomagnesemia at screening	264 subjects randomized 1:1 to treatment with either the magnesium–vitamin B6 combination or magnesium alone	Significant reduction of perceived stress *vs*. baseline; no significant difference between the two treatment groups	(20)
DHA	Stress	6-week double blind randomized placebo-controlled clinical trial on academic personnel, aged 18–60, experiencing stress	93 subjects randomized in an active group treated with fish oil (*n* = 16), a control group treated with olive oil (*n* = 14), or in a control group with no treatment (*n* = 63)	Significant reduction of perceived stress in the active group *vs*. control groups	(21)
DHA, EPA	Burnout	8-week double-blind and placebo-controlled intervention on subjects aged 18–65, with self-reported signs of workplace-related exhaustion	43 subjects randomized in an active group treated with DHA and EPA (*n* = 22), or in a placebo control group (*n* = 21)	Significant improvement of burnout scores and decrease of saliva morning cortisol secretion	(22)
Omega-3 PUFAs	Psychotic disorders	6-month multicenter, double-blind, placebo-controlled trial on subjects aged 13–40, at ultra-high risk for psychotic disorders	304 subjects randomized in an active group treated with n-3 PUFA together with cognitive behavioral case management (CBCM) (*n* = 66) or in placebo group treated with CBCM only (*n*=62)	Increases of the erythrocyte n-3 index and DHA levels are predictive of better symptomatic and functional outcomes at month 6 in the active group	(23)
DHA, EPA	Depression and anxiety	16-week placebo-controlled trial on subjects aged 15–40, with recent onset schizophrenia-spectrum (*n* = 46) or bipolar (*n* = 4) disorders and current psychotic symptoms, treated with risperidone	50 subjects receiving risperidone randomized 1:1 in an active group treated with adjuvant n-3 PUFAs (*n* = 25) or in a placebo control group (*n* = 25)	Substantial decrease in depression-anxiety symptoms in the n-3 PUFA group *vs*. the placebo group	(24)
Omega-3 PUFAs	Major depressive disorder	12-week double-blind, randomized and placebo controlled clinical trial on subjects aged 18–65, with major depressive disorder, treated with sertraline	50 subjects receiving setraline randomized 1:1 in an active group treated with adjuvant n-3 PUFAs (*n* = 25) or in a placebo control group (*n* = 25)	Substantial improvement of symptoms of depression, and of dimensions of anxiety and sleep symptoms in the n-3 PUFA group *vs*. the placebo group	(25)
DHA	Sleep disorder	14–18-week longitudinal, randomized, double-blinded, placebo-controlled trial on pregnant women aged 18–35	48 pregnant women randomized in an active group receiving DHA-supplemented functional food (*n* = 27) or in a placebo group (*n* = 21)	Significant improvement of infant sleep organization in the first 48 h in the DHA group *vs*. the placebo group	(26)
Omega-3+Omega-6 PUFAs,antioxidant vitamins (alpha-tocopherol, gamma-tocopherol, vitamin A)	Mild cognitive impairment	6-month randomized, double-blind, placebo-controlled trial on subjects aged ≥65 years, with probable mild cognitive impairment	46 subjects randomized 1:1 in an active group treated with PUFAs (*n* = 23) or in a placebo group (*n* = 23)	Favorable improvement of functional capacity and cognitive function for the participants receiving the supplementation *vs*. the placebo group	(27)
DHA, EPA	Major depressive disorder	8-week randomized, double-blind, placebo controlled trial on subjects aged 18–80, with major depressive disorder and high plasma levels of inflammation biomarkers	196 subjects randomized in an active group treated with DHA (*n* = 66), an active group treated with EPA (*n* = 65) or in a control group treated with placebo (n=65)	Significant improvement of depressive symptoms in EPA-treated patients with high plasma levels of inflammation biomarkers	(28)
DHA, EPA	Antenatal depressive disorder	12-week randomized trial on pregnant women aged ≥20 years with antenatal depressive disorder	100 subjects randomized in an active group treated with omega-3 PUFAs (*n* = 49) or in a control group treated with placebo (*n* = 51)	Significant association between increased EPA and estradiol and decrease of depressive symptoms in the active group	(29)

### Potential Effects on Anxiety

Anxiety disorders are the most prevalent mental disorders, and produce fear, worry, and a constant feeling of being overwhelmed; they can be triggered by stress conditions and include, among others, panic disorder, agoraphobia, and social anxiety disorder, with a severe impact on the QoL. Several anxiolytic medications, mainly targeting monoamine neurotransmission, have been introduced in the clinical practice for the treatment of anxiety. However, patients may either do not respond to these medications or respond inadequately. Preclinical and clinical studies have shown that supplementation of essential elements can enhance the drugs' anxiolytic effects. The NEURAPRO trial indicated that the administration of omega-3 PUFAs in addition to cognitive behavioral case management could exert therapeutic effects in individuals at ultra-high risk for psychotic disorders with a significant reduction of mood and anxiety disorders (23). These data are consistent with the RCT results obtained by Robinson DG et al. (24), suggesting that an EPA/DHA adjuvant treatment is a potential option for depression and anxiety symptoms in subjects with recent onset psychosis and treated with risperidone (Table 1). Magnesium antagonizes the N-methyl-d-aspartate (NMDA) receptor and exhibits gamma-aminobutyric acid receptor agonist activity; these mechanisms may be at the basis of its anxiolytic activity observed in animal models (30). Consistently, a magnesium-deficient diet contributes to enhancement of anxiety-like behavior in murine models (31), while a systematic review suggests a beneficial effect of magnesium on subjective anxiety, but also recommends additional randomized controlled trials to confirm this observation further (32). Vitamin E deficiency during pregnancy in murine models leads to irreversible alterations in brain glutamate levels and is associated with increased anxiety at adulthood, suggesting the importance of vitamin E during pregnancy to prevent increased anxiety in later life (33).

### Potential Effects on Sleep Disorders

Sleep disorders include, among others, insomnia and obstructive sleep apnea, and cause a disruption of the normal circadian rhythm with a negative impact on physical health and mental well-being. It is well-known that dietary components can directly affect sleep quality and duration (34); hence, a proper modulation of nutrients could positively affect sleep disorders. Some pre-clinical studies showed that omega-3 PUFA deficiency affects the sleep/wake patterns in basal condition and after a peripheral immune challenge, possibly through the dysregulation of cytokine release in cortical neuronal networks (35). A RCT enrolling patients affected by major depressive disorder (MDD) revealed that the intake of adjuvant omega-3 PUFAs in addition to a standard medication improved not only symptoms of depression, but also anxiety and sleep disorders when compared to placebo (25). The intake of a DHA-enriched functional food consumed during pregnancy in a RCT had a beneficial impact on infant sleep organization in the first 48 postnatal hours (26) (Table 1). However, more studies are recommended to further support the positive association between omega-3 PUFA intake and reduction of sleep disorders, as well as to understand the mechanisms by which the modulation of brain omega-3 PUFA levels alters the neuronal circuits underlying sleep/wake states.

### Potential Effects on Mild Cognitive Impairment

MCI has been defined as a transitional stage between healthy aging and dementia, characterized by documented cognitive deficits, together with largely intact daily activities (36). Excessive neuroinflammation triggered by microglia priming associated with aging can affect regions of the brain known to support learning and memory, thus inducing or contributing to MCI. There are currently no approved disease-modifying treatments; consequently, lifestyle modifications, including nutritional habits, have become an important strategy to prevent MCI progression (37). Some nutritional supplements specific for MCI, memory loss and Alzheimer's disease (AD) (e.g., Axona, CerefolinNAC, Souvenaid) are currently marketed and their effectiveness has been analyzed in a variety of meta-analyses, with heterogeneous results (38–40).

An adequate dietary intake of omega-3 PUFAs can slow the age-related MCI and protect against the risk of neurodegenerative dementia (7), and higher levels of plasma EPA+DHA are associated with a lower decline in global cognition and memory (41). In addition, a RCT showed that the supplementation of omega-3+omega-6 PUFAs and antioxidant vitamins, including vitamin E, to older adults affected by MCI induced an improvement of their condition (27) (Table 1). However, a meta-analysis of RCTs indicated the need of longer-term studies to confirm these effects (42). Of interest, a recent RCT including subjects without cognitive impairment but at risk of dementia, treated with 2,152 mg per day of DHA or placebo for over 6 months, indicated that larger doses of DHA are needed for adequate brain bioavailability, as confirmed by analysis of the cerebrospinal fluid (43). Moreover, a stratification of study participants by *APOE4* status revealed its association with the level of delivery of DHA and EPA to the brain before the onset of cognitive impairment. These data, together with the DHA pharmacokinetics characteristics, should be carefully considered when setting up the DHA administration to patients at risk of cognitive impairment, as higher doses of DHA and longer periods of treatment could be necessary to obtain a meaningful remodeling of brain DHA levels. The relationship of plasma vitamin E and markers of vitamin E damage with MCI and AD has been investigated in cognitively normal subjects and in patients, revealing lower levels of tocopherols and vitamin E and increased vitamin E damage in AD and MCI *vs*. control subjects (44). However, vitamin E supplementation to AD patients produced contrasting results (45), suggesting the need for additional studies about this particular issue.

## Depression, Bipolar Disorders, Obsessive-Compulsive Disorder: Which Role for Neuroinflammation?

Neuropsychiatric disorders have been traditionally related to a dysregulation of neurotransmitters such as dopamine, norepinephrine, glutamate, and serotonin, contributing to their mood and cognitive dysfunction aspects. Nonetheless, the persistence of treatment-refractory conditions led to the hypothesis of a relationship between inflammation and neuropsychiatric disorders, supported by the observation that medical conditions associated with chronic inflammatory and immunological abnormalities, including obesity, diabetes, malignancies, rheumatoid arthritis, and multiple sclerosis, are risk factors for depression and bipolar disorder (46, 47). In addition, peripheral immune modulators, such as pro-inflammatory interleukin-1β (IL-1β) and tumor necrosis factor-α (TNF-α), can induce psychiatric symptoms in animal models (11). Finally, peripheral cellular and humoral immunological abnormalities are more prevalent in psychiatric *vs*. healthy control subjects (48–51). Of major interest, prospective studies revealed that high C-reactive protein (CRP) levels can predict a subsequent development of bipolar disorder, while increased levels of CRP or IL-6 are predictive of MDD and psychosis, thus suggesting a causal association between inflammatory status and neuropsychiatric disorders (52, 53).

MDD, bipolar disorders, and obsessive-compulsive disorder (OCD) are complex neuropsychiatric conditions with a set of causes and risks associated with gender, genetic and epigenetic factors, socioeconomic status, lifestyle, stress, alcohol and drug use, and concomitant disease. Some studies suggested a role also for unhealthy Western diet (54). BBB is a highly regulated interface that normally restricts the access to the brain of peripheral inflammatory mediators that could impair the neurotransmission. However, during peripheral inflammatory activation, the permeability of the BBB increases, and may exacerbate or initiate neuropsychiatric and neurological disorders (55). Neuroinflammation represents a reaction of the CNS to events that interfere with tissue homeostasis and is present in virtually all neurological diseases. Microglia function as innate immune cells of the CNS, playing key roles in clearing foreign particles and promoting brain healing after a trauma. Prolonged microglial activation, and in particular of the M1 pro-inflammatory microglia, can contribute to neuroinflammatory responses (56) also through endothelial and BBB dysfunction (57). Moreover, the oxidative stress caused by reactive oxygen/nitrogen species and mitochondrial disarrangements play key roles in neuroinflammation (58, 59). Pro-inflammatory cytokines and high nitric oxide (NO) levels produced by activated microglia may promote reactive oxygen species (ROS) formation (60). Due to its composition and biochemical characteristics, the brain is particularly vulnerable to oxidative stress, able to induce brain lipid peroxidation, damage to membrane phospholipids and to neurotransmitter receptors, and depletion of endogenous antioxidants (61).

A strong relationship between the cytokine system and the neurotransmitter system has been highlighted. A special role in the pathogenesis and somatic consequences of MDD has been described for pro-inflammatory IL-6, whose activity may cause depression through the activation of the hypothalamic-pituitary-adrenal axis or influence of the neurotransmitter metabolism, as summarized elsewhere (62). Within the brain, the immune-related information activates several regions and induces glia cells and neurons to release the same cytokines, in turn acting as neuro-regulators and neurotransmitters (63). In more detail, the link between inflammation and depression is probably based on the glucocorticoid insensitivity caused by inflammation, as well as on the deviation of the essential amino acid tryptophan from the production of serotonin toward the production of kynurenine and of its metabolites (e.g., quinolinic acid and kynurenic acid). The decreased synthesis of serotonin following the tryptophan shunting leads to an increased glutamatergic neurotransmission, known to be associated with a depressed mood (64).

## Depression, Bipolar Disorders, Obsessive-Compulsive Disorder: an Anti-Inflammatory Treatment Approach

By virtue of the relationship between inflammation and neuropsychiatric disorders, increasing attention has been devoted to the therapeutic potential of anti-inflammatory treatments in this setting. Traditional antidepressants can have anti-inflammatory and neuroprotective effects, which might be partly due to their influence on cytokine production, as supported also by the decrease of circulating pro-inflammatory factors (60, 65). However, traditional antidepressants can induce side effects, in particular in case of premature self-discontinuation, and can be ineffective in 30–40% of patients. The enzymes cyclooxygenase-1 (COX-1) and cyclooxygenase-2 (COX-2) are involved in the inflammatory cascade leading to the production of prostacyclines, tromboxanes, and leukotrienes. An increasing number of studies are demonstrating that treatments targeting COX-1 and COX-2 can have a beneficial effect in the subgroup of depressed patients with elevated levels of pro-inflammatory cytokines and in patients affected by other neuropsychiatric disorders (65, 66). In particular, clinical studies concerning the use of non-steroidal anti-inflammatory drugs (NSAIDs) and depression revealed a beneficial effect in some cases, in particular in presence of inflammatory disease comorbidities that, however, has not been confirmed by other studies and needs additional investigation (65).

In addition to drug treatments, lifestyle changes, such as dietary interventions and/or the supplementation of specific micro- and macronutrients could have a beneficial effect on neuropsychiatric disorders, through an action on the immune system (67). Below we summarized the current evidences of the anti-inflammatory activity played by DHA, EPA, magnesium, alpha-tocopherol, and folate in the context of neuroinflammation.

### Focus on EPA, DHA and Neuroinflammation

EPA and DHA have a well-known effect on synthesis, release, receptor function and storage of neurotransmitters during development and in neuropsychiatric disorders (68–71). In reference to inflammatory reactions, generally, DHA, and EPA exert an inhibitory effect on the activation of immune cells from both the innate and the adaptive systems. In particular, omega-3 PUFAs are able to act as signaling molecules and decrease cytokine secretion by macrophages and to suppress inflammasome-mediated inflammation. DHA and EPA are also able to decrease neutrophil migration and increase their phagocytic capacity (72). Of note, during the increase in vascular wall permeability, plasma omega-3 PUFAs reach the site of inflammation, where they are converted to SPM, able to block chronic inflammation and reduce fibrosis (73). A dietary approach including DHA, EPA, folic acid, and vitamin E exhibited beneficial effects on structural and functional recovery in a mouse model of transient middle cerebral artery occlusion, leading to decreased neuroinflammation and improved motor function (74). A national survey conducted in United States indicated a significant association between an EPA+DHA intake in the past 24 h and a reduced prevalence of depressive symptoms by 25% in a sample of 10,480 adults (75). A RCT conducted by Rapaport et al. (28) demonstrated a positive effect of EPA in a subset of subjects affected by MDD and with a high level of inflammatory biomarkers. A RCT conducted in depressed pregnant women revealed that the supplementation with EPA was associated with increased levels of estradiol during pregnancy and together they alleviated the antenatal depression. However, this process was presumably mediated by a mechanism other than anti-inflammation, as the relationship between depressive symptoms and inflammatory cytokines was not significant (29) (Table 1).

### Focus on Vitamin E and Neuroinflammation

Alpha-tocopherol supplementation could modulate oxidative stress and inflammation in Parkinson's disease (PD) or AD, where neuroinflammation plays a major role. However, clear data from RCTs are still missing and indications are still limited mainly to preclinical research. The role of alpha-tocopherol in the brain has been investigated in a murine model with a double-deficiency of vitamins C and E, revealing an increased expression of inflammatory-related genes, possibly causing neuroinflammation in the brain (76). *In vitro* and preclinical *in vivo* studies in a murine model of AD showed that the supplementation of alpha-tocopherol further decreased the neuroinflammation and oxidative stress induced by the candidate drug etodolac (77).

### Focus on Magnesium and Neuroinflammation

Magnesium plays a key role in differentiation, proliferation, functioning, and movement of immune cells and is important for balanced immune cell responses (15, 78). Several studies evaluated serum levels of magnesium in different mental disorders; however, a pathophysiological association between mental disorders and magnesium in patients is not yet fully demonstrated. Some studies in murine models of disease revealed the positive role of magnesium in neuroinflammation reduction (79, 80). The large majority of studies carried out so far in patients have been focused on depression, because of the well-known involvement of magnesium in several core mechanisms of depressive pathophysiology, including inflammation and oxidative stress (81). Among others, investigations proposed that magnesium could relieve depression by blocking the NMDA receptor, whose dysfunction is a major causative factor in depression pathology (82). Additional studies are required to establish the role of magnesium in pathophysiology or treatment of bipolar disorder and OCD.

### Focus on Folic Acid and Neuroinflammation

Nearly 30% of severely depressed inpatients have folate deficiency, accompanied by a functional deficit reflected by increased plasma homocysteine (83). The rise in homocysteine indicates a failure of methylation of homocysteine to methionine, that, in turn, is a precursor of S-adenosylmethionine (SAM), the methyl donor in a number of brain reactions involving neurotransmitters, proteins, nucleoproteins, and membrane phospholipids. Similarly to folic acid, SAM has also been reported to affect mood (83–85). A similar correlation between folate, homocysteine, and disease status has been observed in patients affected by OCD (86). Of interest, hyperhomocysteinemia is associated with neuroinflammation in a murine model of vascular dementia (87). The use of folate-producing probiotic strains can be useful in reduction of neuroinflammation, as indicated by decreased levels of pro-inflammatory cytokines, and by improvement of motor behavior in PD (88). Some RCTs suggest that treatment with folate and vitamin B12 could be helpful in the long-term management of specific populations affected by depressive symptoms. However, the meta-analyses carried out by Almeida OP et al. (89) underlined the low number and heterogeneity of available studies, suggesting additional investigations.

## Discussion

Inadequate intake, systemic diseases, medical therapies, and genetic conditions can lead to deficiencies of specific nutrients, affecting both the central and peripheral nervous systems (3). A new research field, the “Nutritional Psychiatry,” evolved in the last years with the aim to identify the dietary components that are particularly important for mental health, and, where appropriate, involve the prescription of dietary modification/supplementation to prevent or manage disorders (90). In addition, a personalized medicine approach has been proposed in the field of nutrition applied to mental well-being, based on the assessment of specific biomarkers (e.g., nutrient deficiencies, inflammatory cytokine levels, genomic assessment, microbiome analysis) to determine the individual macro- and micronutrient requirements (90). Overall, the research data we reviewed seem to support a positive role for the nutrients EPA, DHA, magnesium, alpha-tocopherol, and folic acid, either alone or in combination with drugs, in the preservation of normal brain function and mental well-being, at least in part through the control of neuroinflammation (Figure 1). Additional clinical trials are warranted to confirm the results obtained so far and gain further insights on the mechanisms of actions and effective dose range of the nutrients EPA, DHA, magnesium, alpha-tocopherol, and folic acid on recovery/preservation of mental health and well-being. In particular, the studies we reviewed suggest that alpha-tocopherol supplementation is not consistently associated with improvements of symptoms of mental disorders. Considering the well-known physiological role played by this nutrient and its lower levels associated with mental disorders (91), we suggest that future adequately powered and properly designed clinical trials should further address this point.

The RCTs we reviewed included subjects of different age (Table 1). An adjustment of nutrients' intake and dosing is necessary in function of age range and of the associated assimilation and metabolism, and of the subjects' health status, with particular reference to elderly people, characterized by changes in physiology, metabolism, and organ function and where presence of comorbidities and/or the accumulation of particular nutrients (e.g., essential metals, as iron, copper, manganese) can contribute to the development of neurodegenerative diseases (92). This aspect should be considered when evaluating the results of RCTs and in the design of future studies focusing on the association between nutrients intake and preservation of mental health.

In addition to the published studies we reviewed, other clinical trials are currently on the way in the setting of nutrients and mental disorders or impairment. Several ongoing trials are focusing in particular on PUFAs: among them, the trial NCT03926351 is investigating the role of high-dose DHA in patients at risk for dementia, while the trial NCT03613844 is further investigating the association between the presence of the APOE ε4 allele and the and the reduced delivery of DHA to the brain. Finally, LO MAPT is a phase III trial currently evaluating the efficacy of omega-3 supplementation on cognitive decline in older adults with low DHA/EPA status and subjective memory complaints or family history of Alzheimer disease (ClinicalTrials.gov Identifier: NCT03691519).

An accurate diagnosis of mental disorders and of their stage, alongside a detailed clinical history is of major importance in clinical trials testing the effectiveness of micro- and macronutrients, in order to enroll more homogeneous cohorts of patients, in turn leading to more reliable study results. Moreover, future research would greatly benefit from the use of more uniform tools to measure both functioning and cognition in patients affected by mental disorders. Finally, a nutritional approach based on multiple agents is recommendable rather than administration of single agent supplements.

## Conclusion

Mental health is an integral and essential component of human health, and an unhealthy lifestyle can be associated with a poor mental health. Scientific findings encourage the integration of micro- and macronutrients in a well-balanced and varied diet, accompanied by a healthy lifestyle, for preservation of normal brain function and well-being. This strategy is of particular importance when considering the global human aging and that the brain suffers significantly from the life-long impact of stress factors.

## Author Contributions

MM drafted the manuscript and approved the submitted version.

## Conflict of Interest

The author declares that the research was conducted in the absence of any commercial or financial relationships that could be construed as a potential conflict of interest.
